# The dermomyotome ventrolateral lip is essential for the hypaxial myotome formation

**DOI:** 10.1186/1471-213X-13-37

**Published:** 2013-10-18

**Authors:** Qin Pu, Aisha Abduelmula, Maryna Masyuk, Carsten Theiss, Dieter Schwandulla, Michael Hans, Ketan Patel, Beate Brand-Saberi, Ruijin Huang

**Affiliations:** 1 Institute of Anatomy, Department of Neuroanatomy, Medical Faculty Bonn, Rheinish Friedrich-Wilhelms-University of Bonn, Bonn, Germany; 2 Institute of Anatomy, Department of Anatomy and Molecular Embryology, Medical Faculty, Ruhr University of Bochum, Bochum, Germany; 3 Institute of Physiology, Medical Faculty Bonn, Rheinish Friedrich-Wilhelms-University of Bonn, Bonn, Germany; 4 School Biological Sciences, University of Reading, Reading, UK; 5 Institute of Anatomy and Cell Biology, Department of Molecular Embryology, Medical Faculty, Albert- Ludwigs-University of Freiburg, Freiburg, Germany

**Keywords:** Somite, Dermomyotome, Myotome, Chicken embryo

## Abstract

**Background:**

The myotome is the primitive skeletal muscle that forms within the embryonic metameric body wall. It can be subdivided into an epaxial and hypaxial domain. It has been shown that the formation of the epaxial myotome requires the dorsomedial lip of the dermomyotome (DML). Although the ventrolateral lip (VLL) of the dermomyotome is believed to be required for the formation of the hypaxial myotome, experimentally evidence for this statement still needs to be provided. Provision of such data would enable the resolution of a debate regarding the formation of the hypaxial dermomyotome. Two mechanisms have been proposed for this tissue. The first proposes that the intermediate dermomyotome undergoes cellular expansion thereby pushing the ventral lateral lip in a lateral direction (translocation). In contrast, the alternative view holds that the ventral lateral lip grows laterally.

**Results:**

Using time lapse confocal microscopy, we observed that the GFP-labelled ventrolateral lip (VLL) of the dermomyotome grows rather than translocates in a lateral direction. The necessity of the VLL for lateral extension of the myotome was addressed by ablation studies. We found that the hypaxial myotome did not form after VLL ablation. In contrast, the removal of an intermediate portion of the dermomyotome had very little effect of the hypaxial myotome. These results demonstrate that the VLL is required for the formation of the hypaxial myotome.

**Conclusion:**

Our study demonstrates that the dermomyotome ventrolateral lip is essential for the hypaxial myotome formation and supports the lip extension model. Therefore, despite being under independent signalling controls, both the dorsomedial and ventrolateral lip fulfil the same function, i.e. they extend into adjacent regions permitting the growth of the myotome.

## Background

The musculature of the body consists of an epaxial and hypaxial component. The epaxial component is composed of the deep back muscles which originate solely from the myotome. In contrast, the hypaxial component includes muscles of the ventrolateral body wall, girdle, limb and tongue. The hypaxial portion yields muscle in different ways. Muscle cells of the limb and tongue muscles and the lateral shoulder girdle muscles are derived from the migrating myogenic precursor cells from the somite. In contrast, the ventrolateral body wall muscles (intercostal and abdominal muscles) and the medial shoulder girdle muscles are formed from the myotome [1-6]. A part of the shoulder girdle muscles, the trapezius and sternocleidomastoideus muscle, originate from the lateral plate mesoderm [7].

The myotome, is the primitive skeletal muscle that forms within embryonic metameric structures called the somite. The somite is initially an epithelial sphere, surrounding a mesenchymal core [8,9]. The mature somite compartmentalizes into a dorsal and a ventral part. The ventral part undergoes an epithelial to mesenchymal transition to form the sclerotome which gives rise to axial cartilages, bones, and tendons [10-12]. The dorsal portion remains as an epithelium and forms a cell sheath, called the dermomyotome, which contributes to the formationof the dorsal dermis and skeletal muscle. The four margins of the dermomyotome fold ventrally and form lip-like borders, called the dorsomedial (DML), ventrolateral (VLL), cranial and caudal dermomyotomal lips. The dermomyotomal lips contribute to myogenic cells in two ways. To form the limb muscle, undifferentiated muscle precursor cells delaminate from the VLL and undergo a long distance migration into the limb bud, where they differentiate into muscle cells [13]. To form the myotome, muscle precursor cells delaminate from all four lips and differentiate into mononuclear myocytes immediately under the dermomyotome [13,14].

The myotome morphogenesis has been extensively studied using the avian model. In a descriptive study using immunohistochemistry staining of myotomal cells, Kaehn et al. suggested that the myotome forms in a medial-to-lateral direction [15]. This model postulates that the oldest myotome cells should be located at the extreme medial margin of the myotome and the youngest at the lateral margin. Based upon fluorescent cell lineage-tracing analysis, however, Denetclaw et al. [16,17] advocated an incremental growth model in which the myotome is predicted grow in the opposite direction. According to this model, new cells are added to the myotome in a lateral-to-medial order. Further observations made by this group showed a two phase model of the myotome formation [18,19]. In the first phase, myocytes from the DML form a thin layer of myotome. In the second phase, new myocytes derived from all four lips are recruited in a superficial-to-deep direction. In contrast, studies using thymidine dating suggested an intercalating growth model for myotome morphogenesis [20-24]. According to this model developed by Kalcheim et al, the first myotomal cells form pioneer cells which serve as a scaffold for the secondary myotomal cells. The pioneer cells translocate first from the DML to the ventral position under the dermomyotome. They then migrate to the cranial border, where they elongate towards the caudal border. The myotome cells of the second wave migrate from both cranial and caudal lip into the myotome between the pioneer cells. Using live imaging, Gros and colleagues provide a comprehensive two step model of myotome formation [25]. In the first step, the myotome is formed by an incremental growth. Cells translocate from the DML and elongate bidirectionally, towards to cranial and caudal border of the dermomyotome. In the second step, the myotome is formed by an intercalating growth. Cells from the cranial and caudal border enter the scaffold made by the first myotomal cells originated from the DML.

Though the myotome is made up of the similar mononuclear myoblast, its epaxial and hypaxial part originate from two different sources and develop through different mechanisms. Cell lineage tracing experiments in chick embryos performed by Denetclaw and Ordahl [17] and Gros et al. [25] showed that the epaxial myotome is derived from the medial half of the somite, while the hypaxial myotome arises from the lateral half of the somite. After ablation of the DML, the medial myotome was truncated. Furthermore, the myotome formation in a DML-ablated somite could be restored by transplantation of a second DML from a donor embryo. Cell lineage tracing revealed that new myotome cells are derived from the donor DML. Based upon these observations Ordahl et al. [26] conclude that the DML drives the dorsal-to-medial growth of the dermomyotome which is essential for the medial extension of the myotome. Eloy-Trinquet and Nicolas characterised the formation of the epaxial and hypaxial myotome and concluded that they originate from distinct cell population [27]. The VLL has always been assumed to execute the same function as the DML during the hypaxial mytome morphogenesis. However, this view is not supported by experimentally evidence. In this study, we analysed the myotome formation after surgical ablation of the VLL in chick embryos. Our findings demonstrate that the VLL is required for the hypaxial myotome formation.

## Results

### The dermomyotome ventrolateral lip grows into the somatopleura

To view the cell movement of the VLL, a GFP-expressing vector was introduced into the lateral part of epithelial somites at the interlimb level of chick embryos of about HH-16 by electroporation. Within the following 24 h the operated embryo developed to about stage 20. Subsequently, movement of GFP-labelled cells was recorded in transversal slices (250 μm) from the embryo using a Time lapse-confocal microscopy for 24 hours. In the beginning of the observation, the VLL had grown into the somatopleura (Additional file 1). The GFP-labelled cells formed a broad cell layer which extended ventrolaterally along the inner side of the somatopleura. The lip was close to the coelom. Based on the observation that the width of the GFP-labelled cell layer increased continuously and the position of its medial border was relatively constant, we propose that the VLL grows actively in a lateral direction rather than being pushed by the intermediate part of the dermomyotome. From a transverse perspective, we showed that some cells from the VLL migrated dorsally to the subectodermal space, while some cells migrated ventrally (Additional file 2).

### Removal of the dermomyotome ventrolateral lip affects the ventrolateral growth of the dermomyotome and myotome

**Figure 1:**
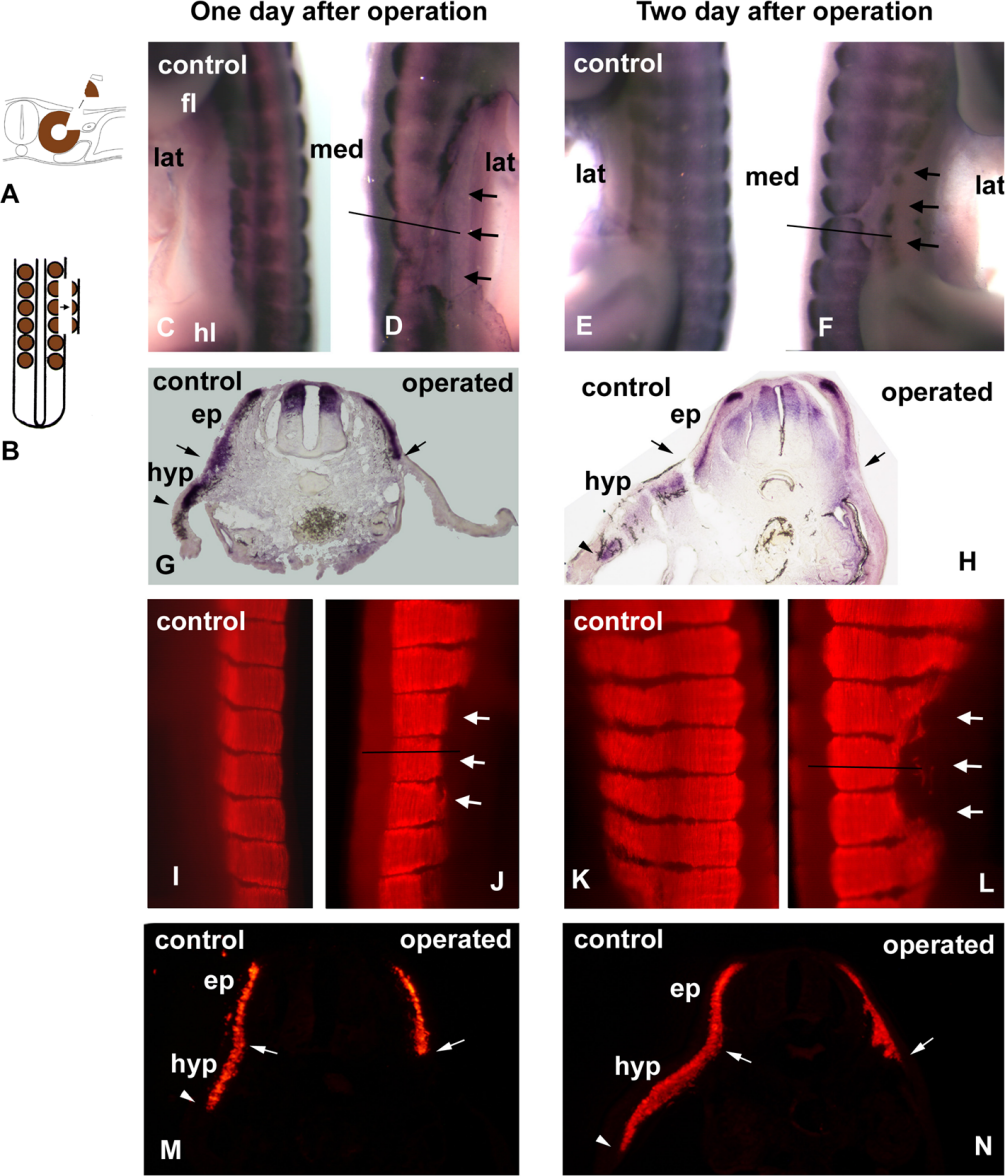
**Ablation of the dorsolateral quarter of epithelial somites (primordium of the ventrolateral dermomyotome lip) interrupted hypaxial myotome growth. A** and **B**: Operation scheme. The dorsolateral quarter of 3 somites (somite III or IV) of a stage 16 chick embryo was ablated. **C**-**F**: *in situ* hybridisation with *Pax3*. **G** and **H**: Transverse section at the level (indicated by a line in **D** and **F**) of the embryo in **D** and **F**. In the operated side the *Pax3* expression was detected only in the epaxial domain (ep), while it was expressed in both epaxial (ep) and hypaxial (hyp) domain of the dermomyotome in the control side. **I**-**L**: Immunohistochemistry with MF20. **M** and **N**: Transverse section at the level (indicated by a line in **J** and **L**) of the embryo in **J** and **L**. In the operated side the myosin heavy chain was detected only in the epaxial myotome (ep), while it was expressed in both epaxial (ep) and hypaxial (hyp) domain of the myotome in the control side. **C**, **D**, **G**, **I**, **J**, **M**: Operated embryos after one day reincubation. **E**, **F**, **H**, **K**, **L**, **N**: Operated embryos after two day reincubation. Operated somites are indicated by arrows (one arrow = one segment) in the whole mount. Arrow heads indicate the ventrolateral end of the hypaxial domain of the dermomyotome and myotome. Arrows mark the border between the epaxial and hypaxial domain. Abbreviations: ep epaxial domain, fl forelimb, hl hind limb, hyp hypaxial domain, lat lateral, med medial, nt neural tube.

To address whether the function of the VLL in the hypaxial myotome formation can be restored by other parts of the dermomyotome, we removed the lateral part of the dermomyotome at the interlimb level at different stages. Since the VLL has invaded the lateral mesoderm at old stages, it is very difficult to identify let alone ablate in embryos after HH-18. Indeed, removal of the most accessible portion of the lateral dermomyotome at HH-18 failed to influence the normal development of the myotome (data not shown). At HH-16/17 the VLL primordium is located just beneath the ectoderm and is easily identified and removed. To ensure that we carried out the ablations robustly, we used in situ hybridisation to gauge the success of our ablation protocols. To that end operated embryos were fixed for *in situ* hybridisation for *Pax3* expression analysis since it is a marker for VLL and DML [3,28-30]. *Pax3* is first expressed uniformly in the entire dermomyotome. As somites mature, the *Pax3*-expression is maintained at a high level in the DML and VLL, but down regulated in central regions. Thus, high level expression of *Pax3* in the hypaxial domain is indicative of the VLL. Ablation of the VLL of single somites only had a mild effect (Additional file 3: Figure S1). A previous study showed that adjacent somitic cells have the capacity to repair regions of ablation [31]. To overcome this hurdle, we ablated three consecutive VLLs. 1 to 2 days after ablation the high level of *Pax3*-expression was no longer detected in the lateral part of the dermomyotome (n = 6, Figure 1). The myotome on the operated side was considerably shorter than on the unoperated regions. Furthermore the most reduced portion was in the centre of the operated side and lesser so at the boundary between operated and unoperated region. In the central region we estimate that the myotome that formed was present at the time of operation and no further myotome has developed (n = 16, Figure 1).

### Cells of the intermediate part of the dermomyotome can not restore the ablated VLL

**Figure 2:**
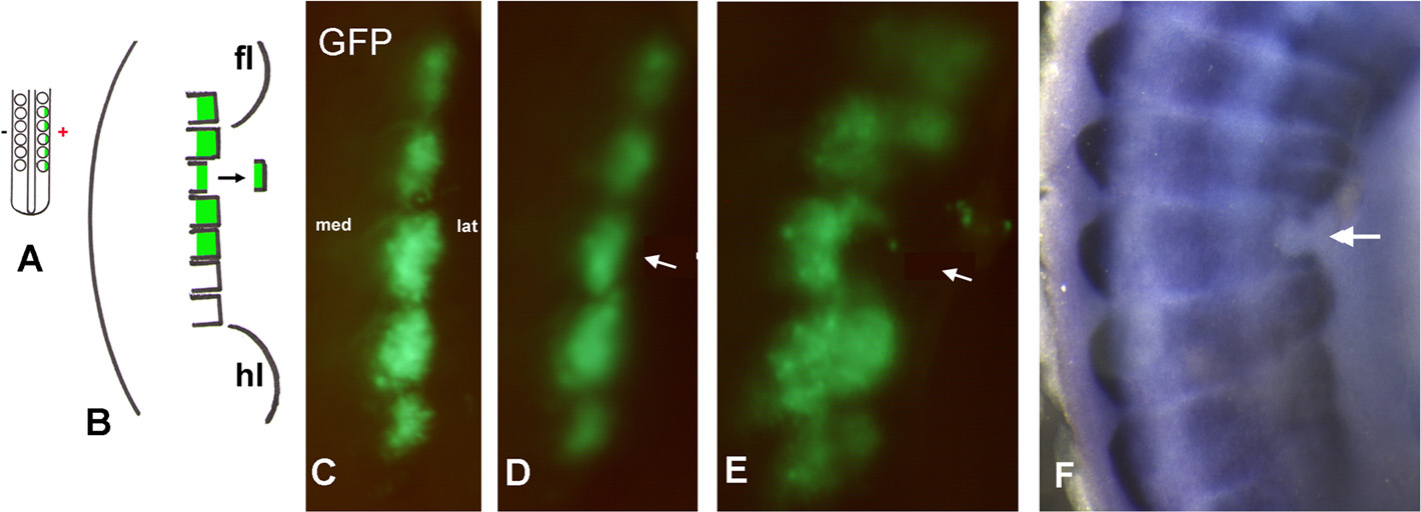
**Ablation of the lateral lip of the dermomyotome after GFP labelling. A** and **B** are scheme of the operation. **A**: The lateral part of interlimb somites was electroporated with a GFP vector. **B**: After one day reincubation, a GFP marked lateral lip was removed. **C-F** are pictures of a same embryo. **C**: One day after the electroporation. The lateral parts of 5 somites were labelled with GFP. **D**: Immediately after the removal of the most lateral part of a somite (arrow). **E**: One day after the ablation. GFP domain of the ablated dermomyotomal lip did not grow as far ventrally as the unoperated somites. **F**: The *Pax3-*expression was affected in the operated segment (arrows). Abbreviations: m medial, l lateral.

It was not known whether cells of the intermediate dermomyotome can support hypaxial myotome extension in the absence of the VLL. To address this question, we labelled the intermediate part of the dermomyotome with GFP, followed by VLL ablation. A GFP-expressing vector was electroporated into the lateral parts of interlimb epithelial somites at HH-16-17 (Figure 2A). Following a reincubation period of 6 hours, a narrow region of GFP could be observed in the lateral somite at the interlimb level. 24 h later the operated embryo had reached HH-19/20 the GFP-positive domain had broadened (Figure 2C). A craniocaudally cut was made in the middle of the GFP-marked dermomyotome part of a segment. Only GFP-positive cells located laterally to the cut were removed. In this way, the VLL was ablated and remaining GFP-positive part represented the ventrolateral edge of the intermediate part. Since the VLL has been invaded into the somatopleura at HH-19/20 and is covered by a very thick tissue layer, as described above, it is very difficult to identify the VLL from the lateral mesoderm tissue without the aid of GFP-labelling. So the VLL ablation was carried out under fluorescence light. To minimize the cell toxicity and bleaching, use of UV light was restricted to short periods to localize GFP-labeled cells and to control whether all GFP- labeled cells were removed. The GFP-labeled cells of the VLL were aspirated with a mouth-controlled glass capillary. The surgical ablation was performed only in one somite, so that the neighbouring somite could serve as control for the operated one. One day after the ablation, we saw the GFP domain of the operated dermomyotome extended less ventrolaterally than that of unoperated dermomyotomes in all embryos (n = 14/14, Figure 2E). These observations indicate that the dermomyotome part located medially to the VLL cannot replace the function of the VLL, with regards to the ventrolateral growth of the dermomyotome.

Embryos from the above manipulation were subsequently analysed for *Pax3* expression. The *Pax3*-expression of the VLL of one embryo (n = 5/5) was missing with the exception of the cranial and caudal edge (Figure 2F). The *Pax3*-expression in the cranial and caudal part of the VLL could be explained due to the incomplete removal of the VLL. Accordingly, the most lateral part of the myotome viewed by the myosin heavy chain antibody (Mf20) was partly disturbed (data not shown).

### Removal of the intermediate part of the dermomyotome epithelium does not significantly affect the myotome formation

Next we ask whether the intermediate part of the dermomyotome epithelium is essential for the growth of the hypaxial myotome. First we investigated the ventrolateral growth of VLL after ablations of the intermediate dermomyotome at the interlimb level. The VLL primordium of interlimb somites was labelled through GFP by electroporation at HH-16/17 as described above. Then, the intermediate part of a dermomyotome was removed. After one to two days of reincubation, the GFP-marked VLL of the operated somites extended as far as those of unoperated somites in all analyzed embryos (n = 8/8, Additional file 4: Figure S2). The VLL was completely normal. The *Pax3*-expression was only partly down-regulated in the intermediate part of the operated dermomyotome. The myotome in the operated somite showed no significant change comparing to the un-operated somites (Additional file 4: Figure S2).

## Discussion

In this study, we investigated the function of the dermomyotome ventrolateral lip (VLL) using surgical ablation technique in the chick embryo. A complication of this procedure is that it is difficult to remove all VLL cells and the remaining cells can reconstitute the ablated tissue. Therefore when small regions are ablated, the phenotype is limited due to regeneration. This can be circumvented by ablating the VLL from consecutive somites. This is a possible explanation why others who have carried out limited ablation experiment have failed to induce changes in myotome growth.

We never observed labelled intermediate dermomyotome moving to the position of the previously ablated VLL. Combined with the observation that the hypaxial myotome truncated after removal of the VLL of 3 somites, we can conclude that the function of the VLL can not be replaced by the intermediate dermomyotome. After removal of the intermediate part, the myotome formed almost normally. It is possible that the remaining cells of the intermediate part could compensate the lost cells, thereby generating enough myocytes for the myotome. These findings would suggest that the intermediate dermomyotome is not essential for the ventrolateral growth of the VLL. The time-lapse data showing the VLL growth supports this interpretation.

The somite contains at least two muscle lineages in its medial and lateral halves, which developmental cell fate is environment dependent [32,33]. The myotomal myogenesis within the medial and lateral somite half occurs in a spatially and temporally different context. The development of the hypaxial myotome is delayed several stages compared to the epaxial myotome [11]. The DML which has been shown to drive the growth and arrangement of the epaxial myotome is always in contact with the surface ectoderm and neural tube during the myotome growth [26]. In contrast, the specification of the hypaxial dermomyotome is induced by lateralising signals from the lateral plate mesoderm/intermediate mesoderm and dorsalising signals from the surface ectoderm [34]. At later stages, the VLL grows into the somatopleura and loses contact with the surface ectoderm. In fact, the VLL is very far away from the ectoderm. The signalling mechanisms controlling the epaxial and hypaxial myotome formation are also different. The medial somite receives signals emanating from the notochord (Shh, Noggin) as well as the neural tube and ectoderm (Wnts, BMPs). These signals coordinate the induction of the epaxial properties of dermomyotome [35-37]. While *Wnt-11* and *En1* are expressed in the epaxial dermomyotome, *Sim1* is expressed in the hypaxial dermomyotome [34,35,38-40]. Though the inducing signal is different, our results show that the VLL is irreplaceable for the formation hypaxial myotome, like the DML [26].

## Conclusions

Our study demonstrates that the dermomyotome ventrolateral lip is essential for the hypaxial myotome formation. Despite being under independent signalling controls, both the dorsomedial and ventrolateral lip fulfil the same function, i.e. they extend into adjacent regions permitting the growth of the myotome.

## Methods

### Embryos

Fertile chick eggs were obtained from the Institute of Animal Science, University of Bonn. The embryos were incubated at 38°C in a humidified atmosphere and staged according to Hamburger and Hamilton (1951) [41]. Ethical approval of the experiments was not needed since all embryos studied here were younger than 10 days.

### Electroporation of a GFP-vector

The electroporation procedure was performed as described by Scaal et al. [42] and Dai et al. [43]. Briefly, electroporations were carried out at the interlimb level of HH-16/17 embryos. GFP-expressing plasmid pCLGFPA was introduced to the lateral part of the epithelial somite.

### Time-lapse microscopy

Time-lapse imaging was performed approximately 24 h after electroporation with a confocal laser scanning microscope (CLSM, Zeiss LSM 510). Embryos were aseptically cut into 200 μm thick horizontal slices with a McIlwain tissue chopper. Slices were attached to collagen-coated (Sigma-Aldrich, C7661) glass coverslips (32mm, Kindler, Freiburg, Germany) fixed by a plasma clot (Sigma, P3266) coagulated with thrombin (Calbiochem, 605157). Tissue slice cultures were used to study cell migration utilizing a Rose-chamber under a Zeiss 10x lens (Plan-Neofluar, NA 0.3). To maintain the incubation settings at 37°C and 5% CO_2_ on the microscope stage, a CTI controller 3700 digital, O_2_ controller, 37-2 digital Tempcontrol, and heating insert P (Zeiss) were used. Additionally the immersion oil objective was heated with a Tempcontrolmini system (Zeiss). For time-lapse imaging of the epithelial somite slices were captured for 10-24 h at 10 min intervals.

### Surgical ablations

Embryo microsurgery was performed as described in previous works with the modification noted below [44-46]. Ablations of the ventrolateral lip (VLL) and intermediate part of the dermomyotome epithelium were performed in similar fashion. For VLL ablations, the dermomyotome epithelium was incised craniocaudally and slightly medial to the VLL. Tissue fragments located laterally to the cut were subsequently removed by mouth aspiration using a glass micropipette. For intermediate part ablations, a second parallel cut was made lateral to the dorsomedial lip (DML). Then the tissue between two cuts was removed. The egg was sealed with tape and reincubated.

### *In situ* hybridization

Operated embryos were fixed in 4% PFA. Whole mount in situ hybridization with *Pax3* was performed as described by Nieto et al. [47]. After photograph, embryos were sectioned (20 μm) using a cryostat microtome.

### Immunohistochemistry

Operated embryos were fixed in Dent’s fixative and then immunohistochemistry stained with the myosin heavy chain monoclonal antibody Mf20 (Developmental Study Hybridoma Bank, Iowa) and GFP polyclonal antibody. Primary Mf20 and GFP antibody were detected with goat-anti-mouse Cy3 and goat-anti-rabbit Cy2, respectively. After photograph, embryos were transversely sectioned in 20 μm thickness using a cryostat microtome.

## Abbreviations

DML: The dorsomedial lip of the dermopmyotome; VLL: The ventrolateral lip of the dermomyotome; GFP: Green fluorescent protein.

## Competing interests

The authors declare that they have no competing interests.

## Authors’ contributions

QP, BB-S, KP and RH designed research. QP, AA, MM, CT, DS, MH and RH performed the experiment. QP, KP and RH wrote the paper. All authors read and approved the final manuscript.

## Supplementary Material

Additional file 1 Live imaging of GFP labelled dermomyotomal ventrolateral lip. Click here for file

Additional file 2 Live imaging of GFP labelled dermomyotomal ventrolateral lip. Click here for file

Additional file 3: Figure S1 Ablation of the dorsolateral quarter of an epithelial somites slightly affected the hypaxial myotome growth. While the *Pax3*-expression was almost completely missing in the position of the VLL of the operated somite after one day of reincubation (n =3), *Pax3*-expression was found to be partly restored in the VLL of the operated segment after two days of reincubation (n = 3). Because *Pax3* was seen only in the cranial and caudal part of the VLL, we presume that cells of the cranial and caudal part of the VLL were not completely removed. So the remaining cells could partly form the VLL after 2 days of reincubation. The technical difficulty of VLL removal of one segment must be the reason for the mildly effect. A and B: Operation scheme. The dorsolateral quarter of a somite (somite III or IV) of a stage 16 chick embryo was ablated. C-F: in situ hybridisation with *Pax3*. C, D: Operated embryos after one day reincubation. E, F: Operated embryos after two day reincubation. Operated somites are indicated by an arrow. Abbreviations: lat lateral, med medial, nt neural tube. Click here for file

Additional file 4: Figure S2 Ablation of the intermediate part of the somite did not affect the dermomyotome and myotome formation. A: Schematic illustration of the operation procedure. 6 Hours after the electroporaion, the lateral part of interlimb somites were labelled through GFP. Then the intermediate part of a GFP-marked dermomyotome was removed. B-C are pictures of a same embryo. B: The GFP positive domain of the operated somite (arrow) elongated as far as the neighbouring somites. C: control side. D: operated side. *Pax3*-expression showed almost normal in the operated segment (arrow). E-H are pictures of a same embryo. E: 6 hours after the electroporation. 6 somites were GFP positive. The intermediate parts of 3 segments were ablated (arrows). Unoperated somites which served as controlled segments were between the operated somite F: After one day of reincubation period, the distribution of GFP cells of the operated somites was not altered. G (control) and H (operated side): Mf20 staining. The lateral growth of the operated myotomes (arrows in H) was comparable with the control segments. Only in one segment (the caudal most one) some myotome cells were missing. Abbreviations: fl forelimb, hl hind limb. Click here for file
