# Correction: The dermomyotome ventrolateral lip is essential for the hypaxial myotome formation

**DOI:** 10.1186/1471-213X-13-41

**Published:** 2013-12-01

**Authors:** Qin Pu, Aisha Abduelmula, Maryna Masyuk, Carsten Theiss, Dieter Swandulla, Michael Hans, Ketan Patel, Beate Brand-Saberi, Ruijin Huang

**Affiliations:** 1 Institute of Anatomy, Department of Neuroanatomy, Medical Faculty Bonn, Rheinische Friedrich-Wilhelms-University of Bonn, Bonn, Germany; 2 Institute of Anatomy, Department of Anatomy and Molecular Embryology, Medical Faculty, Ruhr University of Bochum, Bochum, Germany; 3 Institute of Anatomy, Department of Cytology, Medical Faculty, Ruhr University of Bochum, Bochum, Germany; 4 Institute of Physiology, Medical Faculty Bonn, Rheinische Friedrich-Wilhelms-University of Bonn, Bonn, Germany; 5 School Biological Sciences, University of Reading, Reading, UK; 6 Institute of Anatomy and Cell Biology, Department of Molecular Embryology, Medical Faculty, Albert- Ludwigs-University of Freiburg, Freiburg, Germany

## Correction

Some spelling errors were discovered following the publication of this work [1]. The correct spelling of one of authors name is Dr. Dieter Swandulla and not Dr. Dieter Schwandulla. Accordingly his correct e-mail address is Dieter.Swandulla@ukb.uni-bonn.de. The correct spelling of the University of Bonn is Rheinische Friedrich-Wilhelms-University of Bonn. In addition the current address of Dr. Carsten Theiss is Institute of Anatomy, Department of Cytology, Medical Faculty, Ruhr University of Bochum, Bochum, Germany. We apologise for any inconvenience this may have caused.
